# Attitudes Toward Organ Donation for Persons Who Have a Substance Use Disorder Relative to Other Health Conditions

**DOI:** 10.3389/fpsyt.2021.698645

**Published:** 2021-11-12

**Authors:** Caitlyn J. Grubb, Cecilia L. Bergeria, Andrew S. Huhn, Kelly E. Dunn

**Affiliations:** Johns Hopkins University School of Medicine, Baltimore, MD, United States

**Keywords:** opioid, substance use disorder, organ donation, stigma, overdose

## Abstract

**Background:** Increases in opioid-related overdose and death have led to increases in the number of organs available for donation and transplant, however persons who have a substance use disorder (SUD) may be disadvantaged relative to other health conditions with regard to receiving an organ for transplant.

**Objective:** This study aimed to evaluate perceptions regarding acceptability and priority for organ donation vs. a control condition (resuscitation) for hypothetical persons with nine target health conditions including a substance use disorder, among persons recruited as part of an online survey.

**Methods:** Respondents (*N* = 285; male = 172, female = 113) recruited from Amazon Mechanical Turk rated acceptability and priority that hypothetical persons representing nine target health conditions expected to influence transplant success (including a SUD) receive an organ transplant and resuscitation via a survey hosted by Qualtrics. Primary outcomes of stigma ratings and priority ranking of persons as a function of the hypothetical target health condition were analyzed using Repeated Measures Analyses of Variance and Bonferroni-corrected *t*-tests. Demographic information was presented descriptively for all respondents.

**Results:** Ratings for acceptability and priority for persons who had a SUD were generally lower than ratings for other conditions for both organ for transplant and resuscitation, though respondents reported less stigma toward resuscitation, *F*_(8)_ = 22.35, *p* <0.001 overall. Respondents were least supportive of persons who smoked cigarettes receiving an organ, *p*'s < 0.001. Priority rankings favored persons who were young or had a history of heart disease. Multivariable models determined that target health condition, *F*_(8)_ = 33.64, *p* < 0.001, was a better and more consistent predictor of response than demographic variables that were examined.

**Conclusions:** Data suggest that general perception of acceptability and priority ranking for receipt of life-saving interventions was lower for persons who have a SUD relative to other clinically-relevant health conditions. Research to examine this effect among persons working in the donation system are warranted and efforts to reduce stigma toward persons who have a SUD should be continued.

## Introduction

More than 100,000 persons are currently awaiting an organ donation in the United States (1) and up to 20% of these individuals may die or be removed from the transplant list before a donation is received (2). Donations resulting from fatal drug poisonings appear to have good prognoses. Data suggests donors who experience fatal drug poisonings have lower rates of medical comorbidities than other donor subgroups, and there are no consistent differences in the success of transplants that result from overdose deaths, relative to those who do not (2–7). Despite overdose deaths contributing substantially to the organ donation pool, persons who have a substance use disorder (SUD), are less likely to be placed on an organ donation list or to receive a donation than others (8), and they are often asked to discontinue any substance use disorder-related treatment they may be receiving in order to receive an organ, which threatens their substance-related recovery process (8–11).

The process surrounding decisions for being a transplant recipient is subject to individual decision-making, which presents an opportunity for bias or stigma to influence outcomes and issues such as presence of a SUD are frequently debated (12). Patients must meet specific criteria established by the transplant center and be assigned by a transplant team as a candidate (12). When an organ becomes available, a list is generated that ranks potential transplant candidates based on metrics meant to promote equality and transparency, which include medical factors, time on the waiting list, and distance from the donor and the organ is then offered to the top-ranking candidate (13). In 2018, 10,721 deceased organ donors contributed to 29,676 deceased donor transplants in the United States, and 4,994 organs were discarded (14).

Stigma toward persons who have a SUD, including Opioid Use Disorder (OUD) in particular, has been well-documented within the general population (15) and among healthcare professionals (16–18) and has been shown to profoundly impacts patient access to resources, healthcare, and overall quality of life (19–21). However, general attitudes toward organ transplantation for persons with substance use is largely unknown. This is an important topic to address as this population is contributing large numbers of organs for transplant, but may be disadvantaged in receiving a needed transplant. This initial evaluation provides insight into the degree to which general stigma toward persons with a SUD, and/or OUD in particular, may generalize into settings that could influence critical access to vital organ transplants.

## Materials and Methods

### Respondent Recruitment

The survey was advertised as a “brief health survey” on the Amazon Mechanical Turk (MTurk) platform between 5/2020 and 7/2020. Mturk is a platform used for crowdsourcing, the use of the internet to outsource work to a large sample of people, and is a method for convenience sampling that is increasingly popular (22, 23). Using the MTurk platform, “workers” are paid to complete HITs (human intelligence task) which are posted as jobs on the mTurk website that are completed remotely for which the worker can be paid. Respondents were given a brief eligibility survey and were required to be at least 18 years of age, be located within the United States, and have a prior HIT approval rate >95% to participate in the study. Approval ratings are assigned based on the proportion of previous HITs that were satisfactorily completed relative to all HITs initiated by the worker. In order to participate, workers also must have completed at least one HIT in the past. Respondents were paid $0.10 for the eligibility portion of the survey and those who were admitted into the full study received a bonus of $3.00 for completing the HIT. The mTurk platform allows respondents to complete surveys in a completely anonymous and deidentified manner. Since the survey did not include any protected health or otherwise identifying information, the Johns Hopkins University Institutional Review Board determined this study did not constitute human subjects research.

### Measures

#### Respondent Characteristics

Respondents answered demographic questions that included age, sex, race/ethnicity, education, employment, and marital status. Respondents also indicated whether they were a registered organ donor (yes/no), had experience with organ donation (registered organ donor, organ transplant recipient, were the family member/close friend of recipient, were a living organ donor, were a family/close friend of donor, were a healthcare professional involved with organ donation/transplantation, no involvement), had experience with Alcohol Use Disorder (AUD) or a SUD (e.g., current diagnosis within past year of AUD or a SUD, currently in treatment, in recovery, family/close friend of someone with AUD or a SUD, healthcare professional involved with AUD or a SUD, no experience), had smoked more than 100 cigarettes in their lifetime (yes/no), the average number of cigarettes smoked on days smoked in the past 30 days, and if they were a healthcare worker (yes/no).

#### Stigma

This study used hypothetical scenarios to examine whether persons who had a history of a SUD were systematically considered to be a lower priority for receiving an organ transplant relative to persons who had other chronic illnesses by members of the general public. Eligible respondents responded to a hypothetical questionnaire designed to assess stigma toward hypothetical individuals who represented different chronic health conditions or characteristics that were hypothesized to impact respondent perception of them being an optimal organ transplant recipient. To assess stigma in various situations based on health history and behaviors, respondents were presented with a series of hypothetical people who had various health histories that were hypothesized to impact perceived need for receiving an organ and emergency care (resuscitation). Our measure was adapted from the Attitudes toward Mental Illness Questionnaire, a validated measure for assessing biases toward individuals' various mental illnesses (24). Resuscitation was used as a comparator to organ donation because both represent life-saving, emergency interventions but vary with regard to the rarity of the resource. Hypothetical persons were described as persons who: “ate fast food (McDonald's) everyday,” “had a family history of heart failure (for organ) or had a family history of low blood pressure/hypotension (for resuscitation),” “were in treatment for a SUD,” “had received an organ donation 10 years ago (for organ) or had received resuscitation 3 months ago,” “has Schizophrenia,” “smokes 5 cigarettes a day,” “has maintained sobriety from alcohol for 7 months,” “is 18 years old,” and “is 80 years old” (see Supplementary Material for all vignettes).

Respondents were provided with a brief description of both organ donation and resuscitation (see Supplementary Materials) before indicating how pleased they would be on a scale of 0 (very upset)−10 (very pleased) for the hypothetical individuals to receive each life-saving intervention (organ recipient, resuscitation). All respondents completed ratings for each outcome for each hypothetical condition however the order in which the hypothetical people were presented was randomized within each outcome (organ recipient, resuscitation) to minimize order effects. Respondents were then asked to assign a priority rank to the same hypothetical persons for both organ recipient and resuscitation.

### Data Analysis

Primary outcomes were stigma ratings and priority ranking of persons as a function of the hypothetical target health condition. Demographic information was presented descriptively for all respondents and included age (years), primary employment status (employed vs. not employed), race (white/Caucasian, black/African American, other), never married (yes, no).

Within-subject ratings on the acceptability scale (0–10) and priority rankings (1–9) were compared using a nine (hypothetical condition) × two (organ recipient, resuscitation) repeated-measures analysis of variance (RM-ANOVA). Bonferroni-corrected *t*-tests were used for *post-hoc* pairwise comparisons of acceptability ratings between organ recipient and resuscitation. To model clinical settings and distinguish individuals with stigma from those without stigma, acceptability ratings (0–10) were converted to binary outcomes that were operationalized as evidence of stigma (0 [very upset]-4 [upset]) or no stigma (5 [neutral]-10 [very pleased]), and impact of hypothetical conditions on stigma was compared with binary logistic regression, setting a SUD as the reference variable. Next, multivariate logistic regressions were used to examine the relative contribution of respondent demographic and drug use history on stigma, adjusting for demographic factors hypothesized to impact outcome. Priority rankings were evaluated descriptively and by determining the top three conditions that respondents would prioritize access to organ transplant or resuscitation. Finally, the percent of respondents who showed evidence of stigma and who ranked a target health condition in the top 3 for receiving the outcome of interest were compared using Wilcoxon signed-rank tests. All analyses were conducted using SPSS version 24 and all alpha levels were set to 0.05.

## Results

### Respondents

A total of 440 persons completed the eligibility survey, of which 347 were eligible; 58 were excluded because they did not reside in the U.S. and 35 were excluded because they were 18 years or older. Responses were evaluated to confirm surveys were completed and that respondents had successfully answered attention checks that were embedded throughout the survey, resulting in a final sample of 285 (82.1% of eligible) respondents. Respondent demographics and health characteristics are in Table 1. Respondents were primarily male (60.4%), white/Caucasian (66.3%), and 33.6 (SD = 10.7) years old. Overall, 76.8% of respondents had direct experience with organ donation, either being a registered donor, a recipient or family/close friend of a recipient, or working with persons during the organ donation process. Similarly, 44.2% of persons had direct experience with AUD or a SUD, either being a current or former user, being the family member/close friend of someone with AUD or a SUD, or working with persons who have AUD or a SUD. More than half of respondents had smoked more than 100 cigarettes in their lifetime, and current smokers reported smoking an average of 6.3 (SD = 9.9) cigarettes per smoking day in the past 30 days.

**Table 1:** Respondent characteristics.

	**%, M (SD) (*N* = 285)**
**Demographic**	
Male	60.4
Age (years)	33.6 (10.7)
Living in urban setting	138
**Race**	
White/Caucasian (ref)	66.3
Black/African American	14.7
Other	19.0
**Education**	
High school	6.7
Some college	17.9
College	75.4
Employed	17.2
Never married	38.9
**Health characteristics**	
Work in healthcare industry	40.0
Health insurance	90.8
Direct experience with organ donation^a^	76.8
Registered organ donor	72.5
**AUD or a SUD history**	
Personal experience with AUD or a SUD^b^	44.2
Prior alcohol or substance use	25.9
Smoked >100 cigarettes lifetime	57.5
Number cigarettes smoked per day	6.3 (9.9)

*M, mean; SD, standard deviation, df, degrees of freedom; AUD, alcohol use disorder; SUD, substance use disorder; ^a^Personal experience defined as being the recipient or family member member of a recipient, a living donor or family member of a donor. Participants could select more than 1 option; ^b^Personal experience defined as current diagnosis or treatment, being in recovery, having a family member/friend with AUD or a SUD, or working in the field of AUD or SUD. Participants could select more than 1 option*.

### Acceptability Ratings

**Figure 1:**
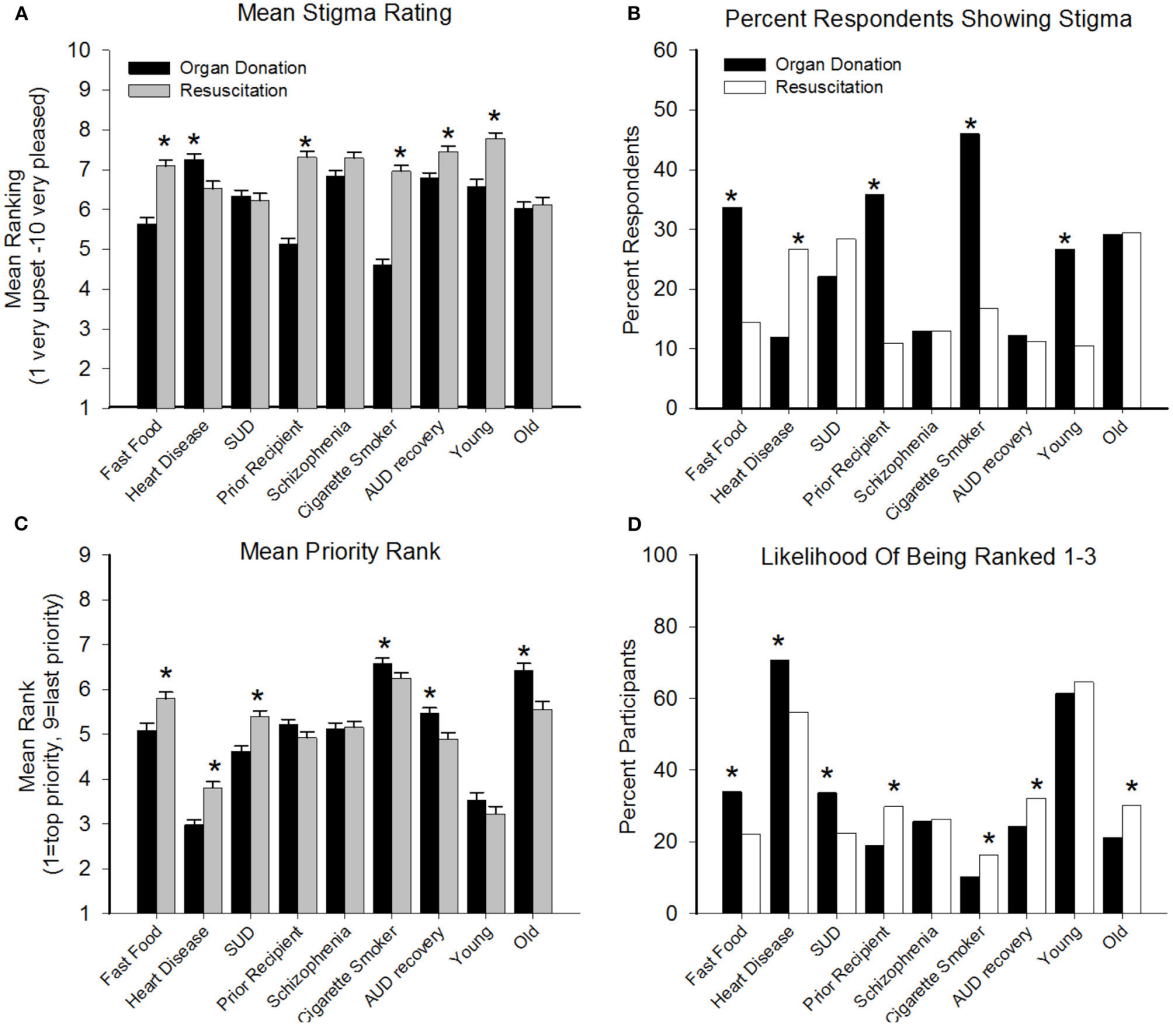
Data present results of acceptability and priority ratings (Y-axes) as a function of the target health conditions (X-axes) with regard to being a recipient of an organ transplant (filled bars) and emergency resuscitation (open/gray bars); dashed lines represent neutral rating threshold or 50% of respondents. (**A**, top left) illustrates mean (SEM) acceptability ratings on a scale of 1 (very upset) to 10 (very pleased), with lower values representing greater stigma. (**B**, top right) illustrates percent of respondents showing evidence of stigma (defined as rating of 0–4) for the target conditions, with higher values representing greater stigma. (**C**, bottom left) illustrates mean (SEM) priority rank from 1 (top priority) to 9 (last priority), with higher numbers representing greater stigma. (**D**, bottom right) illustrates percent of respondents who ranked the target health condition in the top three most worthy of receiving access to outcome. Pairwise comparison of continuous variables **(A,C)** conducted with Bonferroni-corrected *t*-tests and for **(B,D)** with Bonferroni-corrected Wilcoxon signed-rank tests, *denotes significant pairwise comparisons *p* < 0.005. SUD, substance use disorder; AUD, alcohol use disorder.

A two-way RM-ANOVA revealed a significant main effect of hypothetical condition, *F*_(8)_ = 33.64, *p* < 0.001, and a significant hypothetical condition × intervention (organ recipient or resuscitation) interaction effect, *F*_(8)_ = 34.46, *p* < 0.001, on participant's acceptability ratings. Two separate RM-ANOVAs found that acceptability ratings significantly differed depending on whether the hypothetical persons was receiving an organ, *F*_(8)_ = 43.89, *p* < 0.001 or resuscitation, *F*_(8)_ = 22.35, *p* < 0.001. Bonferroni-corrected contrasts indicated hypothetical persons described as “cigarette smokers” were deemed less deserving of an organ relative to all other hypothetical individuals, *p*'s < 0.001 (Figure 1A). Moreover, individuals described as “80 years old” were deemed less deserving of emergency resuscitation compared to other hypothetical individuals, *p*'s < 0.05. Direct comparison of organ recipient and resuscitation revealed respondents were generally more likely to support resuscitation relative to an organ donation for all hypothetical persons, *F*_(1)_ = *38.43, p*< *0.001*, with the exception that persons described as “had previously had a heart transplant” were rated more positively for receiving an organ transplant than other hypothetical persons.

Significantly more respondents showed evidence of stigma (yes/no) toward persons who ate fast food, were prior recipients, were cigarette smokers, and who were 18 years old when the target was organ donation vs. resuscitation. Only one target condition, a family history of heart failure/low blood pressure, resulted in significantly more stigma toward resuscitation relative to organ donation *(p's*< *0.05)* (Figure 1B). The highest stigma (close to 50%) was observed for persons who smoked cigarettes that were being considered for organ donation.

### Prioritization of Treatment Access

A two-way RM-ANOVA revealed a significant main effect of hypothetical person, *F*_(8)_ = *602.53, p*< *0.001* and a significant hypothetical person × intervention (organ recipient or resuscitation) interaction effect, *F*_(8)_ = *55.77, p*< *0.001*, on priority rankings (Figure 1C). Bonferroni-corrected contrasts revealed significantly lower rankings (representing higher priority) for organ donations relative to resuscitation for persons eating fast food, heart disease, or a SUD, and significantly lower rankings for resuscitation relative to organ donation for persons who were cigarette smokers, in AUD recovery, or 80 years old *(p's* < 0.05*)*.

Comparison of the percent of respondents ranking each condition in the top three priority rating revealed that relative to resuscitation, persons who eat fast food, had heart disease, and had a SUD were more frequently prioritized for organ donation (Figure 1D). Significantly more respondents prioritized individuals who were prior organ recipients, cigarette smokers, in recovery for AUD, and 80 years old for receiving resuscitation relative to organ donation *(p's*< *0.05)*. More than 50% of respondents rated persons with heart disease and who were 18 years old in the top three rankings for both organ transplant and resuscitation, and no other hypothetical persons achieved more than 50% endorsement for either intervention.

### Correlates of Stigma

The logistic regression evaluation of acceptability of organ donation was significant, $\chi_{\left(8\right)}^{2}$ = *515.69, p*< *0.001* and revealed that, relative to someone who had a SUD, respondents showed significantly more stigma toward persons who had a history of heart disease and significantly less stigma toward all other hypothetical persons with the exception that being in recovery from AUD did not differ from persons who had a current SUD (Table 2). When access to resuscitation was examined, a logistic regression revealed that persons with a SUD had significantly lower acceptability ratings relative to all other hypothetical persons (Table 2).

**Table 2:** Univariate evaluation of stigma as a function of health condition.

	**Organ donation**			**Resuscitation**		
	**Wald (df = 1)**	**OR (95% CI)**	***p*-value**	**Wald (df = 1)**	**OR (95% CI)**	***P*-value**
**Target health condition**						
Substance use disorder	ref	ref	ref	ref	ref	ref
Fast food	47.267	0.28 (0.19–0.40)	<0.001	4.684	1.49 (1.04–2.12)	0.03
Heart transplant	28.377	2.56 (1.81–3.61)	<0.001	100.442	6.46 (4.49–9.31)	<0.001
Prior recipient	13.698	1.88 (1.35–2.64)	<0.001	177.661	22.10 (14.00–39.90)	<0.001
Schizophrenia	87.123	7.50 (4.91-11.50)	<0.001	176.841	21.4 (13.60-33.50)	<0.001
Cigarette smokers	84.449	7.04 (4.64–10.70)	<0.001	171.7	18.10 (11.70–27.90)	<0.001
Recovery from AUD	1.579	1.24 (0.89–1.72)	0.21	156.629	13.30 (8.89–20.00)	<0.001
18 years old	9.251	0.59 (0.43–0.83)	0.002	11.227	1.83 (1.28–2.60)	0.001
80 years old	35.176	2.89 (2.03–4.10)	<0.001	178.397	23.00 (14.50–36.40)	<0.001

*OR, odds ratio; AUD, alcohol use disorder; df, degrees of freedom; ref, reference category*.

Multivariate models (Table 3) indicated that acceptability ratings toward organ donation and resuscitation were driven primarily by the hypothetical health condition rather than respondent-level demographic and/or drug use characteristics. The multivariate regression model for organ donation ratings found that being female, unmarried, working in the healthcare industry, having a personal history of AUD or a SUD, and being a current smoker (as well as smoking more than 5 cigarettes a day) were the only general characteristics significantly associated with stigma ratings, whereas the hypothetical health conditions produced robust and significant associations with acceptability ratings. This same general pattern was observed for resuscitation, wherein the only general characteristics associated with stigma ratings were being female and having a personal history of AUD or a SUD, while the hypothetical health conditions remained significantly associated with stigma ratings.

**Table 3:** Multivariate evaluation of stigma as a function of target health condition.

	**Organ donation**			**Resuscitation**		
	**Wald (df = 1)**	**AOR (95% CI)**	***P*-value**	**Wald (df = 1)**	**AOR (95% CI)**	***P*-value**
**Demographic**						
Male	3.79	1.12 (0.99-1.49)	0.05	5.00	0.78 (0.63–0.97)	0.03
Age (years)	1.46	1.01 (0.99–1.02)	0.23	0.09	1.00 (0.99–1.01)	0.77
Living in urban setting	2.64	0.84 (0.69–1.04)	0.10	3.05	0.82 (0.66–1.03)	0.08
**Race**						
White/Caucasian (ref)	ref	ref	ref	ref	ref	ref
Black/African American	3.75	0.77 (0.58–1.00)	0.053	1.00	1.34 (0.99–1.82)	0.06
Other	0.001	1.04 (0.79–1.28)	0.10	0.75	1.14 (0.88–1.49)	0.32
**Education**						
High school	ref	ref	ref	ref	ref	ref
Some college	0.56	0.85 (0.55–1.32)	0.46	3.57	0.77 (0.48–1.25)	0.29
College	2.13	0.75 (0.51–1.11)	0.15	1.13	0.66 (0.43–1.02)	0.06
Employed	0.60	0.91 (0.7001.17)	0.44	0.02	1.02 (0.77–1.35)	0.88
Never married	7.71	0.71 (0.56–0.91)	0.01	3.40	0.79 (0.61–1.02)	0.07
**Health characteristics**						
Work in healthcare industry	3.40	0.81 (0.65–1.01)	0.07	0.44	0.92 (0.73–1.17)	0.51
Health insurance	0.17	1.07 (0.77–1.48)	0.68	0.01	0.99 (0.69–1.41)	0.94
Direct experience with organ donation	0.00	0.99 (0.72–1.36)	0.96	1.99	1.28 (0.91–1.79)	0.16
Registered organ donor	2.04	0.83 (0.65–1.07)	0.15	0.17	1.94 (0.72–1.25)	0.68
**AUD or SUD history**						
Personal experience with AUD or a SUD	16.50	0.55 (0.41–0.73)	0.00	10.56	1.70 (1.23–2.34)	0.00
Prior AUD/SUD use	12.52	0.60 (0.45–0.80)	0.00	7.37	1.55 (1.13–2.13)	0.01
Smoked >100 cigarettes lifetime	3.37	0.79 (0.61–1.02)	0.07	0.34	1.09 (0.82–1.44)	0.56
Smoking >5 cigarettes per day	4.35	1.01 (1.00–1.03)	0.04	0.75	1.01 (0.99–1.02)	0.39
**Target health condition**						
Substance Use Disorder (ref)	ref	ref	ref	ref		ref
Fast food	49.94	0.26 (0.18–0.37)	0.00	4.81	1.50 (1.04–2.15)	0.028
Heart Transplant	30.26	2.72 (1.91–3.89)	0.00	103.01	6.82 (4.71–9.88)	0.00
Prior Recipient	14.63	1.97 (1.39–2.79)	0.00	181.97	23.88 (15.06–37.87)	0.00
Schizophrenia	92.43	8.43 (5.46–13.02)	0.00	181.11	23.03 (14.59–36.37)	0.00
Cigarette Smokers	89.60	7.89 (5.15–12.11)	0.00	175.81	19.49 (12.56–30.23)	0.00
Recovery from AUD	1.69	1.25 (0.89–1.76)	0.19	160.36	14.29 (9.47–21.57)	0.00
18 years old	9.85	0.58 (0.41–0.81)	0.00	11.54	1.86 (1.3–2.66)	0.001
80 years old	37.48	3.10 (2.16–4.46)	0.00	182.73	24.79 (15.56–39.48)	0.00

*AOR, adjusted odds ratio; df, degrees of freedom; AUD, alcohol use disorder; SUD, substance use disorder*.

## Discussion

This study provides insight into attitudes toward providing life-saving interventions in the form of organs donations or emergency resuscitation for hypothetical persons representing different and clinically-meaningful health conditions. The study compared general acceptability ratings for organ donation recipients vs. resuscitation, as these both represent life-saving interventions but vary with regard to scarcity of the resource. These data suggest that relative to other hypothetical conditions, persons who have a SUD (i.e., Nicotine Use Disorder) had generally greater stigma directed toward them with regard to organ donation and resuscitation efforts, though the latter was less pronounced. This effect persisted in a multivariable model that suggested the greatest driver of stigma ratings was the stated hypothetical health condition rather than individual-level respondent characteristics. Priority rankings revealed a similar effect, whereby persons who had a SUD were rarely ranked as a top-3 recipient for organ donation or resuscitation.

These data provide initial evidence that the well-documented stigma toward persons who have a SUD may extend to provision of life-saving interventions. Interestingly, the highest observed stigma for organ donation (but not resuscitation) was toward persons who smoked cigarettes, followed by persons who ate fast food or who had already received an organ. A similar trend was observed with regard to priority rankings, wherein persons who smoked cigarettes or were older were rated as the lowest priority for organ donation. Acceptability and priority ratings for receiving resuscitation was generally better than for organ donation across all outcomes. These data suggest that respondents may have had general stigma toward persons who had a SUD, independent of the scarcity of the intervention being proposed. Multivariable analysis suggested that target health condition was the primary driver of acceptability and priority ratings, with few additional demographics contributing to the model. The one exception was persons who had AUD or a SUD or had a personal history of AUD or a SUD, which was positively associated with acceptability ratings for both organ donation and resuscitation interventions. The fact that being in recovery from AUD was the only hypothetical condition that did not differ significantly from a SUD further emphasizes that a personal history of AUD or a SUD may influence willingness to provide interventions to those individuals.

Stigma toward persons who have a SUD in healthcare settings is well-documented and prior studies have repeatedly found that medical care was more likely to be suboptimal or avoidant for persons being treated for a SUD relative to other patients, resulting in greater feelings of mistrust from the patient, withholding of pain medication, shorter office visits, and teams vs. individual provider visits (25–28). Although stigma is always problematic, the notion that it has the potential to impact decisions regarding the delivery of crucial life-saving interventions supports additional research in this area. Stigma has also been reported with regard to prescribing of the life-saving medication naloxone (Narcan) to persons with OUD, which could have more proximal consequences than what was evaluated here (29).

The fact that no respondent-level demographic or other variables were consistently associated with stigma suggested that the hypothetical condition was driving the majority of ratings. This is a positive outcome, because it suggests that efforts to reduce stigma toward a health condition could be effective (17, 30, 31), particularly in healthcare settings (17, 25, 32). Short Brief Intervention and Training (SBIRT) programs with healthcare providers have also been shown to produce enduring reductions in stigma toward persons who have a SUD (33, 34). SBIRT interventions may be optimal for medical settings because their short time commitment increases feasibility of delivery (35).

This study is limited by its simplistic presentation of hypothetical persons to members of the general public. This was considered an appropriate first step to determine the need for additional research, but it is recognized that the organ donation process is highly complex and dynamic and that future studies that more specifically target persons involved in the organ donation process and that provide more realistic decision-making scenarios are necessary. An additional limitation is that data were collected remotely via the MTurk crowdsourcing platform, which prevents objective verification of respondent information. Studies have found mTurk workers to be younger, more educated, less healthy and are more likely to be depressed, however empirical comparisons have found the results from mTurk workers, including populations of substance users, to be as valid and reliable as field and laboratory experiments. This concern is somewhat mitigated by our focus on members of the general public as opposed to a specific clinical subpopulation. Further, while convenience sampling limits our ability to estimate the prevalence of attitudes in the general populations, our analyses are informative because we focused on whether certain demographics predicted stigmatized attitudes.

## Conclusion

Overall, the number of organs available for donation are increasing as a function of drug-related poisonings however persons who have a SUD experience greater stigma than do persons who do not have a SUD and that stigma may influence attitudes regarding their appropriateness for receiving an organ transplant. This study extended prior work by suggesting that persons identified as having a SUD were considered by members of the general public to be less deserving of receiving life-saving treatments, including both organ donation and emergency resuscitation, relative to persons representing other hypothetical health conditions that were also hypothesized to similarly impact the success of those interventions. Since the decision-making process for organ donation is somewhat subjective, it remains susceptible to stigma and bias and these data suggest that additional studies that more specifically assess attitudes and perceptions among persons directly involved in the organ donation decision-making process are warranted, to determine whether these general attitudes extend to those life-saving settings. In that regard, these data are a first step in this line of research and support the development of additional studies to more directly examine stigma toward persons who have a SUD in the organ donation process.

## Data Availability Statement

The raw data supporting the conclusions of this article will be made available by the authors, without undue reservation.

## Ethics Statement

The studies involving human participants were reviewed and approved by JHUSOM IRB. Written informed consent for participation was not required for this study in accordance with the national legislation and the institutional requirements.

## Author Contributions

CG, CB, and KD developed and managed the study. All study authors contributed to the data analyses and manuscript preparation.

## Funding

This study was supported in part by NIDA grants R01DA035246, R01DA042926, and R01DA040644.

## Conflict of Interest

The authors declare that the research was conducted in the absence of any commercial or financial relationships that could be construed as a potential conflict of interest.

## Publisher's Note

All claims expressed in this article are solely those of the authors and do not necessarily represent those of their affiliated organizations, or those of the publisher, the editors and the reviewers. Any product that may be evaluated in this article, or claim that may be made by its manufacturer, is not guaranteed or endorsed by the publisher.
