# Supplementary material for: Attitudes Toward Organ Donation for Persons Who Have a Substance Use Disorder Relative to Other Health Conditions
Source: Front Psychiatry. 2021 Nov 12;12:698645. doi: 10.3389/fpsyt.2021.698645 (PMC8633394; doi:10.3389/fpsyt.2021.698645)
Supplement: Supplementary file 1 [file Image_1.PDF]

AMIQ_John

John has been INJECTING HEROIN daily for 1 year. Please select the answer that best reflects your views for each statement.

	Strongly Agree	Agree	Neither Agree nor Disagree	Disagree	Strongly Disagree	Don't Know
Will this damage John's career?	◯	◯	◯	◯	◯	◯
I would be comfortable if John was my colleague at work?	◯	◯	◯	◯	◯	◯
I would be comfortable about inviting John to a dinner party?	◯	◯	◯	◯	◯	◯

John has been INJECTING HEROIN daily for 1 year. Please select the answer that best reflects your views for each statement.

	Very likely	Quite Likely	Neither Likely nor Unlikely	Quite Unlikely	Very Unlikely	Don't Know
How likely do you think it would be for John's wife to leave him?	◯	◯	◯	◯	◯	◯
How likely do you think it would be for John to get in trouble with the law?	◯	◯	◯	◯	◯	◯

AMIQ_Mark

Mark is a practicing CHRISTIAN. He attends church every Sunday and attempts to lead a Christian life. Please select the answer that best reflects your views for each statement.

	Strongly Agree	Agree	Neither Agree nor Disagree	Disagree	Strongly Disagree	Don't Know
Will this damage Mark's career?	○	○	○	○	○	○
I would be comfortable if Mark was my colleague at work?	○	○	○	○	○	○
I would be comfortable about inviting Mark to a dinner party?	○	○	○	○	○	○

Mark is a practicing CHRISTIAN. He attends church every Sunday and attempts to lead a Christian life. Please select the answer that best reflects your views for each statement.

	Very likely	Quite Likely	Neither Likely nor Unlikely	Quite Unlikely	Very Unlikely	Don't Know

	Very likely	Quite Likely	Neither Likely nor Unlikely	Quite Unlikely	Very Unlikely	Don't Know
How likely do you think it would be for Mark's wife to leave him?	○	○	○	○	○	○
How likely do you think it would be for Mark to get in trouble with the law?	○	○	○	○	○	○

AMIQ_Michael

Michael has SCHIZOPHRENIA. He needs an injection of medication every 2 weeks. He was detained in hospital for several weeks 2 years ago because he was hearing voices from the Devil and thought that he had the power to cause earthquakes. He has been detained under the Mental Health Act 1983 in the past. Please select the answer that best reflects your views for each statement.

	Strongly Agree	Agree	Neither Agree nor Disagree	Disagree	Strongly Disagree	Don't Know
Will this damage Michael's career?	○	○	○	○	○	○

	Strongly Agree	Agree	Neither Agree nor Disagree	Disagree	Strongly Disagree	Don't Know
I would be comfortable if Michael was my colleague at work?	○	○	○	○	○	○
I would be comfortable about inviting Michael to a dinner party?	○	○	○	○	○	○

Michael has SCHIZOPHRENIA. He needs an injection of medication every 2 weeks. He was detained in hospital for several weeks 2 years ago because he was hearing voices from the Devil and thought that he had the power to cause earthquakes. He has been detained under the Mental Health Act 1983 in the past. Please select the answer that best reflects your views for each statement.

	Very likely	Quite Likely	Neither Likely nor Unlikely	Quite Unlikely	Very Unlikely	Don't Know
How likely do you think it would be for Michael's wife to leave him?	○	○	○	○	○	○
How likely do you think it would be for Michael to get in trouble with the law?	○	○	○	○	○	○

AMIQ_Robert

Robert is a convicted CRIMINAL. He has spent time in prison for several convictions for theft and shoplifting and is currently ON BAIL for fraud and burglary. Please select the answer that best reflects your views for each statement.

	Strongly Agree	Agree	Neither Agree nor Disagree	Disagree	Strongly Disagree	Don't Know
Will this damage Robert's career?	○	○	○	○	○	○
I would be comfortable if Robert was my colleague at work?	○	○	○	○	○	○
I would be comfortable about inviting Robert to a dinner party?	○	○	○	○	○	○

Robert is a convicted CRIMINAL. He has spent time in prison for several convictions for theft and shoplifting and is currently ON BAIL for fraud and burglary. Please select the answer that best reflects your views for each statement.

	Very likely	Quite Likely	Neither Likely nor Unlikely	Quite Unlikely	Very Unlikely	Don't Know
How likely do you think it would be for Robert's wife to leave him?	○	○	○	○	○	○
How likely do you think it would be for Robert to get in trouble with the law?	○	○	○	○	○	○

AMIQ_Steve

Steve has been DRINKING HEAVILY for 5 years. He is now going for TREATMENT and has started attending Alcoholics Anonymous meetings. Please select the answer that best reflects your views for each statement.

	Strongly Agree	Agree	Neither Agree nor Disagree	Disagree	Strongly Disagree	Don't Know
Will this damage Steve's career?	○	○	○	○	○	○
I would be comfortable if Steve was my colleague at work?	○	○	○	○	○	○

	Strongly Agree	Agree	Neither Agree nor Disagree	Disagree	Strongly Disagree	Don't Know
I would be comfortable about inviting Steve to a dinner party?	○	○	○	○	○	○

Steve has been DRINKING HEAVILY for 5 years. He is now going for TREATMENT and has started attending Alcoholics Anonymous meetings. Please select the answer that best reflects your views for each statement.

	Very likely	Quite Likely	Neither Likely nor Unlikely	Quite Unlikely	Very Unlikely	Don't Know
How likely do you think it would be for Steve's wife to leave him?	○	○	○	○	○	○
How likely do you think it would be for Steve to get in trouble with the law?	○	○	○	○	○	○

AMIQ_Peter

Peter has DIABETES. He needs to inject insulin every day and has a special diet. Please select the answer that best reflects your views for each statement.

	Strongly Agree	Agree	Neither Agree nor Disagree	Disagree	Strongly Disagree	Don't Know
Will this damage Peter's career?	○	○	○	○	○	○
I would be comfortable if Peter was my colleague at work?	○	○	○	○	○	○
I would be comfortable about inviting Peter to a dinner party?	○	○	○	○	○	○

Peter has DIABETES. He needs to inject insulin every day and has a special diet. Please select the answer that best reflects your views for each statement.

	Very likely	Quite Likely	Neither Likely nor Unlikely	Quite Unlikely	Very Unlikely	Don't Know
How likely do you think it would be for Peter's wife to leave him?	○	○	○	○	○	○

	Very likely	Quite Likely	Neither Likely nor Unlikely	Quite Unlikely	Very Unlikely	Don't Know
How likely do you think it would be for Peter to get in trouble with the law?	○	○	○	○	○	○

AMIQ_Tim

Tim is DEPRESSED and took a large amount of acetaminophen (Tylenol) last month to TRY TO HURT HIMSELF. Please select the answer that best reflects your views for each statement.

	Strongly Agree	Agree	Neither Agree nor Disagree	Disagree	Strongly Disagree	Don't Know
Will this damage Tim's career?	○	○	○	○	○	○
I would be comfortable if Tim was my colleague at work?	○	○	○	○	○	○
I would be comfortable about inviting Tim to a dinner party?	○	○	○	○	○	○

Tim is DEPRESSED and took a large amount of acetaminophen (Tylenol) last month to TRY TO HURT HIMSELF. Please select the answer that best reflects your views for each statement.

	Very likely	Quite Likely	Neither Likely nor Unlikely	Quite Unlikely	Very Unlikely	Don't Know
How likely do you think it would be for Tim's wife to leave him?	○	○	○	○	○	○
How likely do you think it would be for Tim to get in trouble with the law?	○	○	○	○	○	○

Instructions

The following questions will contain medical and other life scenarios. Please answer HONESTLY how you would feel for each scenario, and make decisions assuming you are not strictly following specific rules or regulations. Your answers will remain anonymous.

Organ Donation Scenarios

These questions will ask your thoughts about ORGAN DONATION. Please consider the following information about organ donations when making your decisions.

- A large team of professionals from several medical areas discuss whether a patient may be eligible for consideration of a transplant.
- Patients must pass a series of medical tests, support structure questions, and interviews asking how well they would be able to take care of the new organ.
- Patients who pass this process are then placed on an organ waiting list.
- Patients wait for an organ until they need it most or are determined to have the best chance of survival after receiving the organ.
- Patients are matched with the best organ based on several medical factors, organ size, and physical location.
- Not everyone that needs an organ will get one.

Assume the following patients need an ORGAN TRANSPLANT. Assume all patients equally need the transplant and family support, financial, and geography

aspects are the same. The only thing that makes the patients different is the information given below.

Please rate your feelings about each outcome on a scale of VERY UPSET to VERY PLEASED. Assume each outcome has no effect on the other outcomes listed in this section.

	Very Upset		Upset			Neutral		Pleased		Very Pleased	
	0	1	2	3	4	5	6	7	8	9	10
Patient D.E. previously received a heart transplant 10 years ago and does NOT receive a transplant now.											
Patient F.G. smokes 5 cigarettes a day and does NOT receive a transplant.											
Patient A.B. eats McDonald's everyday and receives a transplant.											

	Very Upset	Upset	Neutral	Pleased	Very Pleased
	0 1	2 3	4 5 6	7 8	9 10
Patient H.I. is an 18 year old and does NOT receive a transplant.					
Patient G.H. has maintained sobriety from alcohol for 7 months and receives a transplant.					
Patient E.F. has schizophrenia and receives a transplant.					
Patient B.C. has a family history of heart failure and receives a transplant.					
Patient C.D. is in a treatment program for substance use disorder and receives a transplant.					

	Very Upset		Upset		Neutral		Pleased		Very Pleased	
	0	1	2	3	4	5	6	7	8	9	10
Patient I.J. is an 80 year old and receives a transplant.											

Please rank the order in which YOU would prioritize the patients mentioned above to receive an organ transplant. To rank, select the item you wish to move and drag it to the desired location on the list.

- Patient A.B. eats McDonald's everyday.
- Patient B.C. has a family history of heart failure.
- Patient C.D. is in a treatment program for substance use disorder.
- 4 Patient D.E. previously received a heart transplant 10 years ago.
- Patient E.F. has schizophrenia.
- Patient F.G. smokes 5 cigarettes a day.
- Patient G.H. has maintained sobriety from alcohol for 7 months.
- Patient H.I. is an 18 year old.
- Patient I.J. is an 80 year old.

Are there any scenarios in which you think a person should NOT receive an organ transplant?

Which of the following is your favorite food? This is a data quality check. Regardless of your true preference, please select Spaghetti.

- Pizza
- Spaghetti
- Ice cream
- Candy

COVID Scenarios

The following questions will ask your thoughts about VENTILATION. Please consider the following information

about VENTILATION when making your decisions.

-COVID-19 is a respiratory infection caused by the coronavirus (SARS-CoV-2) and can make it difficult to breathe.
-A ventilator is used to provide oxygen to the body when the body otherwise couldn't.

Assume the following patients have COVID-19 and need to be put on one of the FEW VENTILATORS in a hospital. Assume all patients equally need the ventilator and family support, financial, and geography aspects are the same. The only thing that makes the patients different is the information given below.

Please rate your feelings about each outcome on a scale of VERY UPSET to VERY PLEASED. Assume each outcome has no effect on the other outcomes listed in this section.

Very Upset		Upset			Neutral		Pleased		Very Pleased	
0	1	2	3	4	5	6	7	8	9	10

	Very Upset	Upset	Neutral	Pleased	Very Pleased

0 1 2 3 4 5 6 7 8 9 10

Patient C.D. is in a treatment program for substance use disorder and gets a ventilator.

Patient G.H. has maintained sobriety from alcohol for 7 months and does NOT get a ventilator.

Patient A.B. eats McDonald's everyday and does NOT get a ventilator.

Patient B.C. has a family history of asthma and gets a ventilator.

Patient I.J. is an 80 year old and gets a ventilator.

	Very Upset	Upset	Neutral	Pleased	Very Pleased
	0 1	2 3	4 5 6	7 8	9 10
Patient D.E. was previously treated for COVID-19 and gets a ventilator now.					
Patient E.F. has schizophrenia and gets a ventilator.					
Patient F.G. smokes 5 cigarettes a day and does NOT get a ventilator.					
Patient H.I. is an 18 year old and gets a ventilator.					

Please rank the order in which YOU would prioritize the patients with COVID-19 mentioned above to be put on a ventilator. To rank, select the item you wish to move and drag it to the desired location on the list.

Patient H.I. is an 18 year old.

Patient G.H. has maintained sobriety from alcohol for 7 months.

Patient B.C. has a family history of asthma.

Patient C.D. is in a treatment program for substance use disorder.

Patient A.B. eats McDonald's everyday.

Patient D.E. was previously treated for COVID-19.

Patient F.G. smokes 5 cigarettes a day.

Patient I.J. is an 80 year old.

Patient E.F. has schizophrenia.

Are there any scenarios in which you think a person should NOT be treated for COVID-19?

Emergency Care Scenarios

These questions will ask your thoughts about RESUSCITATION. Please consider the following information about RESUSCITATION when making your decisions.

- Resuscitation is preformed when a person's heart stops beating.
- Resuscitation is also called CPR.
- Resuscitation includes chest compression and sometimes breathing into the person's airway.

Assume the following people need RESUSCITATED by a paramedic. Assume all people equally need the resuscitation and family support, financial, and geography aspects are the same. The only thing that makes the patients different is the information given below.

Please rate your feelings about each outcome on a scale of VERY UPSET to VERY PLEASED. Assume each outcome has no effect on the other outcomes listed in this section.

Very Upset		Upset			Neutral		Pleased			Very Pleased
0	1	2	3	4	5	6	7	8	9	10

	Very Upset	Upset	Neutral	Pleased	Very Pleased
	0 1	2 3	4 5 6	7 8	9 10
Patient G.H. has maintained sobriety from alcohol for 7 months and gets resuscitated.					
Patient H.I. is an 18 year old and gets resuscitated.					
Patient I.J. is an 80 year old and does NOT get resuscitated.					
Patient C.D. is in a treatment program for substance use disorder and does NOT get resuscitated.					
Patient A.B. eats McDonald's everyday and gets resuscitated.					

	Very Upset	Upset	Neutral	Pleased	Very Pleased
	0 1	2 3	4 5 6	7 8	9 10
Patient D.E. was previously resuscitated 3 months ago and gets resuscitated now.					
Patient F.G. smokes 5 cigarettes a day and gets resuscitated.					
Patient E.F. has schizophrenia and gets resuscitated.					
Patient B.C. has a family history of low blood pressure (hypotension) and does NOT get resuscitated.					

Please rank the order in which YOU would prioritize the patients mentioned above to be resuscitated. To rank,

select the item you wish to move and drag it to the desired location on the list.

- Patient F.G. smokes 5 cigarettes a day.
- Patient H.I. is an 18 year old.
- Patient D.E. was previously resuscitated 3 months ago.
- Patient I.J. is an 80 year old.
- Patient B.C. has a family history of low blood pressure (hypotension).
- Patient A.B. eats McDonald's everyday.
- Patient G.H. has maintained sobriety from alcohol for 7 months.
- Patient E.F. has schizophrenia.
- Patient C.D. is in a treatment program for substance use disorder.

Are there any scenarios in which you think a person should not be resuscitated?

Which of the following words begins with the letter B ? This is a data quality check. Regardless of your true preference, please select Brown.

- Brown
- Bottoms
- Blue
- Bleach

Stimulus Check

These questions will ask your thoughts about the federal STIMULUS CHECK. Please consider the following information about STIMULUS CHECK when making your decisions.

-The stimulus check was provided to US Citizens by the Federal Government in response to the Coronavirus pandemic.
-The check could be worth as much as $1200 per adult and $500 additional per dependent child.

Assume the following people are eligible to receive an EXTRA STIMULUS CHECK due to COVID-19. Assume all people equally need the money and family support, financial, and geography aspects are the same. The only thing that makes the patients different is the information given below.

Please rate your feelings about each outcome on a scale of VERY UPSET to VERY PLEASED. Assume each outcome has no effect on the other outcomes listed in this section.

	Very Upset	Upset	Neutral	Pleased	Very Pleased

0 1 2 3 4 5 6 7 8 9 10

Person H.I. is an 18 year old and does NOT receive an extra check.

Person F.G. smokes 5 cigarettes a day and does NOT receive an extra check.

Person E.F. has schizophrenia and receives an extra check.

	Very Upset	Upset	Neutral	Pleased	Very Pleased
	0 1	2 3	4 5 6	7 8	9 10
Person B.C. has a family history of cancer and receives an extra check.					
Person G.H. has maintained sobriety from alcohol for 7 months and receives an extra check.					
Person I.J. is an 80 year old and receives an extra check.					
Person A.B. eats McDonald's everyday and does NOT receive an extra check.					
Person D.E. previously received an extra stimulus check and receives an extra check now.					

	Very Upset	Upset	Neutral	Pleased	Very Pleased
	0 1	2 3	4 5 6	7 8	9 10
Person C.D. is in a treatment program for substance use disorder and receives an extra check.					

Please rank the order in which YOU would prioritize the people mentioned above to receive an extra stimulus check due to COVID-19. To rank, select the item you wish to move and drag it to the desired location on the list.

- Person E.F. has schizophrenia.
- Person F.G. smokes 5 cigarettes a day.
- Person I.J. is an 80 year old.
- Person A.B. eats McDonald's everyday.
- Person H.I. is an 18 year old.
- Person C.D. is in a treatment program for substance use disorder.
- Person B.C. has a family history of cancer.

Person D.E. previously received an extra stimulus check.

Person G.H. has maintained sobriety from alcohol for 7 months.

Are there any scenarios in which you think a person should not receive an extra stimulus check?

Job

The following questions will ask your thoughts about HIRING SOMEONE for an entry-level job opportunity. Assume that all training will be provided on the job and that you regularly hire people who have no prior experience in this area.

Assume you are looking to HIRE SOMEONE for a job opportunity. The following people have the same

qualifications for the job you are hiring for. Assume all people equally need the job and family support, financial, and geography aspects are the same. The only thing that makes the people different is the information given below.

Please rate your feelings about each outcome on a scale of VERY UPSET to VERY PLEASED. Assume each outcome has no effect on the other outcomes listed in this section.

	Very Upset		Upset			Neutral		Pleased		Very Pleased	
	0	1	2	3	4	5	6	7	8	9	10
Person I.J. is an 80 year old and gets the job.											
Person G.H. has maintained sobriety from alcohol for 7 months and does NOT get the job.											
Person B.C. has a family history of kidney disease and does NOT get the job.											

	Very Upset	Upset	Neutral	Pleased	Very Pleased

0 1 2 3 4 5 6 7 8 9 10

Person E.F. has schizophrenia and gets the job.

Person A.B. eats McDonald's everyday and gets the job.

Person C.D. is in a treatment program for substance use disorder and gets the job.

Person F.G. smokes 5 cigarettes a day and does NOT get the job.

Person H.I. is an 18 year old and gets the job.

Person D.E. was fired from your company 5 years ago and gets the job <u>now</u>.

Please rank the order in which YOU would prioritize the people mentioned above to get the job. To rank, select the item you wish to move and drag it to the desired location on the list.

- Person C.D. is in a treatment program for substance use disorder.
- Person A.B. eats McDonald's everyday.
- Person G.H. has maintained sobriety from alcohol for 7 months.
- Person B.C. has a family history of kidney disease.
- Person H.I. is an 18 year old.
- Person F.G. smokes 5 cigarettes a day.
- Person D.E. was fired from your company 5 years ago.
- Person E.F. has schizophrenia.
- Person I.J. is an 80 year old.

Are there any scenarios in which you think a person should not receive a job you are hiring for?

Which of the following is your favorite sport? This is a data quality check. Regardless of your true preference, please select Soccer.

- Basketball
- Soccer
- Volleyball
- Baseball

Demographics

What would you consider to be your PRIMARY race?

- White/Caucasian
- Black/African American
- Native Indian
- Asian
- Native Hawaiian/Pacific Islander
- More than 1 race

○ [ ] Other (please specify)

○ Prefer not to answer

Do you have any Hispanic background?

○ Yes, I AM Hispanic

○ No, I AM NOT Hispanic

○ Prefer not to answer

What is your current MARITAL STATUS?

○ Never Married

○ Married

○ Remarried

○ Divorced

○ Widowed

○ Prefer not to answer

About how much money did your household make (including employment wages, SSI, disability, etc...) in the past year?

[▾]

Do you have health insurance?

Which best describes your experience with organ donation? (Select all that apply)

- ☐ Registered organ donor
- ☐ Organ transplant recipient
- ☐ Family member/close friend of organ transplant recipient
- ☐ Living organ donor
- ☐ Family/close friend of an organ donor
- ☐ Healthcare professional involved with organ donation/transplantation
- ☐ Other
- ☐ No involvement with organ donation

Are you a registered organ donor?

- ◯ Yes
- ◯ No
- ◯ Not sure

Which best describes your experience with alcohol and/or substance use disorder? (Select all that apply)

- ☐ Current diagnosis (within the past year) of alcohol and/or substance use disorder
- ☐ Currently in treatment for alcohol and/or substance use disorder
- ☐ In recovery from an alcohol and/or substance use disorder
- ☐ Family/close friend of someone with an alcohol and/or substance use disorder
- ☐ Healthcare professional involved with alcohol and/or substance use disorder
- ☐ ______ Other
- ☐ No experience with alcohol and/or substance use disorder

You indicated that you have a current diagnosis, in current treatment, or in recovery from alcohol and/or substance use disorder.

	Which of the following substances have you used in your lifetime more than once? Check if yes	Which of the following substances would you say caused the biggest problems? Check if yes
Alcohol	☐	○
Marijuana/Cannabis	☐	○
Opioids/heroin/fentanyl	☐	○

	Which of the following substances have you used in your lifetime more than once? Check if yes	Which of the following substances would you say caused the biggest problems? Check if yes
Cocaine/Crack	☐	○
Other Stimulants/methamphetamine	☐	○
Hallucinogens	☐	○
Other ________	☐	○

Which best describes your experience with the healthcare profession? (Select all that apply)

- ☐ Current healthcare professional (Please write your job title, doctor, nurse, counselor etc.) ________
- ☐ Former/retired healthcare professional
- ☐ Family member/close friend of a healthcare professional
- ☐ ________ Other
- ☐ No experience with the healthcare profession

If your lifetime, have you smoked at least 100 or more cigarettes (total)?

- Yes
- No

In the past 30 days, on how many days have you smoked any cigarette?

On those days, how many cigarettes have you normally smoked (on AVERAGE)?

How soon after you wake up do you smoke your first cigarette?

- Within 5 minutes
- 5-10 minutes
- 31-60 minutes
- More than 60 minutes

Addiction is a(n) (Select all that apply):

- ☐ Disease
- ☐ Brain disease
- ☐ Behavioral disorder
- ☐ Personal weakness
- ☐ Lack of willpower
- ☐ Medical problem
- ☐ Mental health problem
- ☐ Easily curable problem
- ☐ Personal choice
- ☐ Other (please specify)

End of survey and QC questions

Thank you for completing this survey. Did you experience any computer, phone, or other technical problems while you were completing the survey?

- ○ Yes- I had problems
- ○ No- I did NOT have problems

Should we keep and use your data?

Please say "YES" if you answered the questions to the best of your ability.

Please say "NO" if you did not answer the questions accurately, or if something else happened that prevented the majority of responses from being accurate. This will NOT STOP you from being bonused but will help us know whether to include your data in our analyses.

- ◯ Yes- Keep and use these data
- ◯ No- something went wrong, don't use these data

Is there anything more you'd like to tell us about this survey, about your experience with alcohol or substance use disorder, or about your knowledge or interest in organ donation? We appreciate any and all feedback you may have.

If you have no comments, please write "N/A"

Please enter your MTurk Worker ID

Please return to MTurk and the following number to receive your bonus: ${e://Field/mTurkCode}

Powered by Qualtrics
